# Protection by Means of Perinatal Oral Sodium Thiosulfate Administration against Offspring Hypertension in a Rat Model of Maternal Chronic Kidney Disease

**DOI:** 10.3390/antiox12071344

**Published:** 2023-06-26

**Authors:** You-Lin Tain, Chih-Yao Hou, Guo-Ping Chang-Chien, Sufan Lin, Chien-Ning Hsu

**Affiliations:** 1 Department of Pediatrics, Kaohsiung Chang Gung Memorial Hospital, Kaohsiung 833, Taiwan; tainyl@cgmh.org.tw; 2 Institute for Translational Research in Biomedicine, Kaohsiung Chang Gung Memorial Hospital, Kaohsiung 833, Taiwan; 3 College of Medicine, Chang Gung University, Taoyuan 330, Taiwan; 4 Department of Seafood Science, National Kaohsiung University of Science and Technology, Kaohsiung 811, Taiwan; chihyaohou@webmail.nkmu.edu.tw; 5 Center for Environmental Toxin and Emerging-Contaminant Research, Cheng Shiu University, Kaohsiung 833, Taiwan; guoping@csu.edu.tw (G.-P.C.-C.);; 6 Institute of Environmental Toxin and Emerging-Contaminant, Cheng Shiu University, Kaohsiung 833, Taiwan; 7 Super Micro Mass Research and Technology Center, Cheng Shiu University, Kaohsiung 833, Taiwan; 8 Department of Pharmacy, Kaohsiung Chang Gung Memorial Hospital, Kaohsiung 833, Taiwan; 9 School of Pharmacy, Kaohsiung Medical University, Kaohsiung 807, Taiwan

**Keywords:** thiosulfate, hydrogen sulfide, asymmetric dimethylarginine, gut microbiota, hypertension, chronic kidney disease, developmental origins of health and disease (DOHaD)

## Abstract

Hydrogen sulfide (H_2_S) and related reactive sulfur species are implicated in chronic kidney disease (CKD) and hypertension. Offspring born to CKD-afflicted mothers could develop hypertension coinciding with disrupted H_2_S and nitric oxide (NO) signaling pathways as well as gut microbiota. Thiosulfate, a precursor of H_2_S and an antioxidant, has shown anti-hypertensive effects. This study aimed to investigate the protective effects of sodium thiosulfate (STS) in a rat model of maternal CKD-induced hypertension. Before mating, CKD was induced through feeding 0.5% adenine chow for 3 weeks. Mother rats were given a vehicle or STS at a dosage of 2 g/kg/day in drinking water throughout gestation and lactation. Perinatal STS treatment protected 12-week-old offspring from maternal CKD-primed hypertension. The beneficial effects of STS could partially be explained by the enhancement of both H_2_S and NO signaling pathways and alterations in gut microbiota. Not only increasing beneficial microbes but maternal STS treatment also mediates several hypertension-associated intestinal bacteria. In conclusion, perinatal treatment with STS improves maternal CKD-primed offspring hypertension, suggesting that early-life RSS-targeting interventions have potential preventive and therapeutic benefits, awaiting future translational research.

## 1. Introduction

Reactive sulfur species (RSS) have emerged as important molecules in redox regulation and have significant roles in health and disease [1,2]. Various biochemical forms of RSS are closely linked biochemically, including hydrogen sulfide (H_2_S), iron–sulfur clusters, sulfane sulfur, etc. [3]. 

The production of H_2_S can occur via three pathways—enzymatic, non-enzymatic, and bacterial origins. H_2_S is synthesized from L-cysteine via three enzymes, which are cystathionine γ-lyase (CSE), cystathionine β-synthase (CBS), and 3-mercaptopyruvate sulfurtransferase (3MST) [4]. H_2_S can also be produced in the gastrointestinal tract by sulfate- reducing bacteria (SRB), which use reduced compounds as a source of energy, reducing sulfate to H_2_S [5]. Additionally, non-enzymatic H_2_S production occurs through sulfane sulfur.

Thiosulfate, belonging to the sulfane sulfur family, is a major oxidation product of H_2_S. On the other hand, thiosulfate can be reduced to recreate H_2_S. Thiosulfate has been clinically used in the form of sodium thiosulfate (STS). Its indications include calciphylaxis, carbon monoxide toxicity, acute cyanide poisoning, and cisplatin toxicities [4]. In addition to being an H_2_S donor, STS has antioxidant and anti-inflammatory properties. Accordingly, STS has become a potential treatment candidate for several diseases [6]. 

An estimated 10% of people have chronic kidney disease (CKD) [7]. As CKD can originate in early life through so-called renal programming [8], a superior strategy to improve kidney health worldwide is to avert, not just treat, kidney disease. CKD is reported to influence up to 3–4% of women of reproductive age [9]. Maternal CKD is intimately tied to adverse outcomes of pregnancy and the health of the offspring [10]. Previously, we observed that adult rats born from dams with CKD develop hypertension, which perinatal L-cysteine supplementation prevented [11]. The beneficial actions of cysteine are accompanied by a restoration of H_2_S signaling, a reduction of oxidative stress, and the alteration of gut microbiota composition [11].

As an H_2_S donor as well as an antioxidant, STS treatment has revealed benefits against kidney disease and hypertension in several animal models [12,13,14]. Given this background, we hypothesize that STS treatment during gestation and lactation can prevent offspring hypertension induced by maternal CKD. The protective mechanisms of maternal STS treatment were also evaluated.

## 2. Materials and Methods

### 2.1. Animal Experiments

All animal experiments were conducted with approval from the Institutional Animal Ethics Committee at our hospital (Permit #2020110202); the procedures were consistent with the recommendations of the Care and Use of Laboratory Animals of the National Institutes of Health and following Animal Research: Reporting of In Vivo Experiments (ARRIVE) guidelines. Timed-pregnant Sprague Dawley (SD) rats were obtained from BioLASCO Taiwan Co. Ltd. (Taipei, Taiwan) for breeding. Upon arrival at our AAALAC-accredited animal facility, rats were housed individually in cages provided with standard laboratory chow and tap water ad libitum. 

We used an established model of maternal CKD consisting of feeding with chow containing 0.5% adenine protein to the dam for three weeks before gestation as previously described [15]. At 11 weeks old, female rats were mated. The day of copulatory plug detection was designated as gestational day 0. We randomly divided the dams into one of four treatments (*n* = 3 per group): a normal diet (ND), a diet containing 0.5% adenine (CKD), a normal diet with STS (NDST), and a diet containing 0.5% adenine with STS (CKDST). STS was orally administered in drinking water at a dosage of 2 g/kg/day during gestation and lactation. The dosage and route chosen rely on previous studies in rats [13,14]. Following parturition, litters from each dam were culled to eight pups to maintain consistency in pup growth. As males are more likely to be hypertensive than females [16], only male offspring were included in the experiment. 

BP was determined using the CODA rat tail-cuff system (Kent Scientific Corporation, Torrington, CT, USA) in offspring over time at ages ranging from 3 to 12 weeks. To ensure accuracy and reproducibility, the rats were acclimated to restraint and tail-cuff inflation for one week before the measurement. For each rat, five measurements were recorded at each time point. Three stable consecutive measures were taken and averaged [14]. A total of 32 rats (*n* = 8 per group) were sacrificed at 12 weeks of age. Before sacrifice, fresh fecal samples were collected in the morning and stored at −80 °C. Rats were anesthetized using an intraperitoneal injection of xylazine (10 mg/kg) and ketamine (50 mg/kg), then euthanized with an intraperitoneal overdose of pentobarbital. Kidneys were removed, and the cortex and inner medulla were then dissected and snap-frozen in liquid nitrogen. Kidney samples were stored at −80 °C. Blood samples were collected using heparin tubes.

### 2.2. NO Parameters

Several biochemical parameters of the NO pathway were determined via Agilent 1100 HPLC (Santa Clara, CA, USA) with the OPA-3MPA derivatization reagent [14]. Plasma concentrations of L-arginine and symmetric and asymmetric dimethylarginine (SDMA and ADMA, inhibitors of NO synthase) were analyzed in duplicate. The L-arginine-to-ADMA ratio was calculated to denote NO bioavailability [17].

### 2.3. Plasma H_2_S and Thiosulfate

We used a validated method using HPLC–Mass Spectrometry to measure H_2_S and thiosulfate, as described previously [11]. The HPLC system (Agilent Technologies 1290) was coupled to an Agilent 6470 Triple Quadrupole LC/MS and an electrospray ionization source. The solvent system consisted of water and acetonitrile with 0.1% formic acid and an eluent flow rate of 300 µL/min was used. We measured thiosulfate derivative pentafluorobenzyl (PFB)-S_2_O_3_H and H_2_S derivative sulfide dibimane (SDB). Phenyl 4-hydroxybenzoate (PHB) was utilized as an internal standard. Selected reaction monitoring mode was utilized to detect target compounds with a targeted *m*/*z* 212.99 → 93, *m*/*z* 415 → 223, and *m*/*z* 292.99 → 81, for PHB, SDB, and PFB-S_2_O_3_H, respectively. The intra-assay variability for H_2_S and thiosulfate was 4% and 6%, respectively.

### 2.4. H_2_S-Producing Enzymes

Western blotting was performed according to our earlier report [18]. Renal cortex tissues were homogenized, and equal amounts of protein were loaded into each well (200 µg per gel well). After transferring from gel to membrane, Ponceau S staining (PonS, Sigma-Aldrich, Darmstadt, Germany) was applied as a total protein normalization method to detect all sample proteins. Antibodies used to detect H_2_S-producing enzymes are listed in Table 1. Quantitative integrated optical density (IOD) analysis of the Western blot densitometry band was performed through Quantity One Analysis software version 4.6.3 (Bio-Rad, Hercules, CA, USA). The relative protein abundance was presented as the IOD/PonS to correct protein loading variations. 

### 2.5. 16S rRNA Gene Sequencing and Analysis

As we described previously, metagenomic DNA was isolated from frozen fecal samples. V1–V9 full-length 16S gene sequencing and analysis were performed at the Biotools Co., Ltd. (New Taipei City, Taiwan) [18]. PCR amplification was performed with barcoded 16S gene-specific primers for multiplexed SMRTbell library (PacBio, Menlo Park, CA, USA) preparation and sequencing procedure. The QIIME2 was applied to analyze data from high-throughput 16S rRNA sequencing [19]. From the amplicon sequence variant (ASV) sequences, a phylogenetic tree was formed via FastTree (QIIME2).

Sequencing analysis included alpha and beta diversity analysis and different taxa analysis. As alpha diversity indices, Faith’s phylogenetic diversity (PD) index and Shannon index were utilized to determine the microbiota richness and evenness. Beta diversity analysis was conducted based on principal coordinate analysis (PCoA) with unweighted UniFrac distance and Analysis of Similarities (ANOSIM) for comparison of the differences in bacterial composition between groups. Linear discriminant analysis effect size (LEfSe) difference analysis was applied to find differentially abundant taxa [20].

### 2.6. Statistics

Quantitative data are presented as means ± the standard error of the mean (SEM). Statistical analyses were conducted with one-way ANOVA. A *p*-value less than 0.05 was considered statistically significant, and Tukey’s post hoc test was applied if the *p*-value was less than 0.05. OIIME2 was performed to generate phylogenetic beta diversity, and further to perform PCoA using the R program based on unweighted Unifrac distance. LEfSe used the two-tailed nonparametric Kruskal–Wallis test to evaluate the significance of differences in ASVs in 2 groups. A set of pairwise tests among 2 groups was performed using the unpaired Wilcoxon test. Finally, linear discriminant analysis (LDA) was performed to estimate the effect size of each differentially abundant taxa. For stringency, the gut microbiotas were considered significantly different if their differences had a *p*-value < 0.05 and an LDA score (log10) > 4. Statistical analysis was carried out using SPSS (SPSS Inc., Chicago, IL, USA).

## 3. Results

### 3.1. Offspring Outcomes

**Figure 1:**
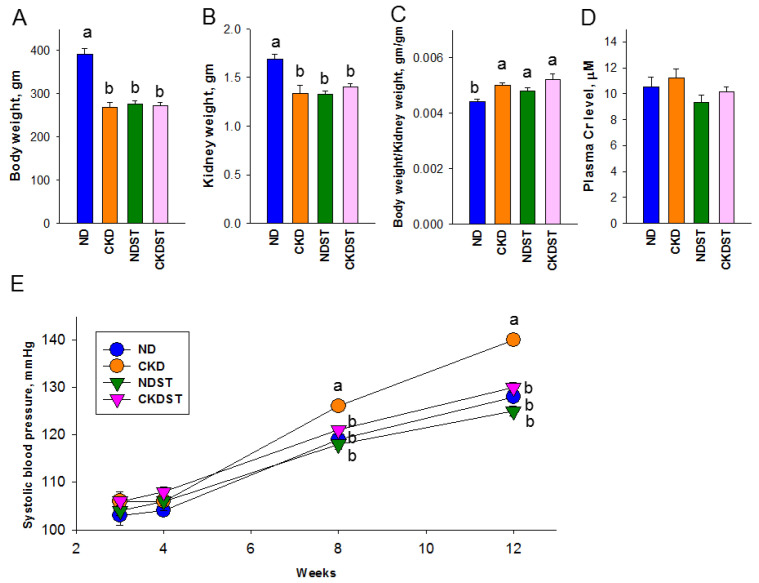
Offspring (**A**) body weight, (**B**) kidney weight, (**C**) body-weight-to-kidney-weight ratio, (**D**) plasma creatinine (Cr) level, and (**E**) systolic blood pressure. N = 8/group. Different letters above the column show significant differences between groups.

We observed no difference in offspring in terms of mortality, sex ratio, or litter size between the four treatments. The offspring born to dams treated with the adenine diet or STS weighed significantly less than their control counterparts (Figure 1A). A similar pattern was observed for kidney weight (Figure 1B). However, the kidney weight to body weight ratio was lowest in the ND group compared to others (Figure 1C). The plasma concentration of creatinine was comparable between the four groups (Figure 1D). Systolic blood pressure (SBP) in offspring, measured via the tail-cuff method at different ages, is presented in Figure 1E. Maternal CKD elicited a rise in SBP during 8–12 weeks of age, which maternal STS treatment prevented. Collectively, these findings indicated that maternal CKD induced hypertension, renal hypertrophy, and low body weights in adult progeny. Maternal STS administration similarly caused renal hypertrophy and low body weights in normal control offspring but prevented maternal CKD-induced offspring hypertension. 

### 3.2. H_2_S Pathway

**Figure 2:**
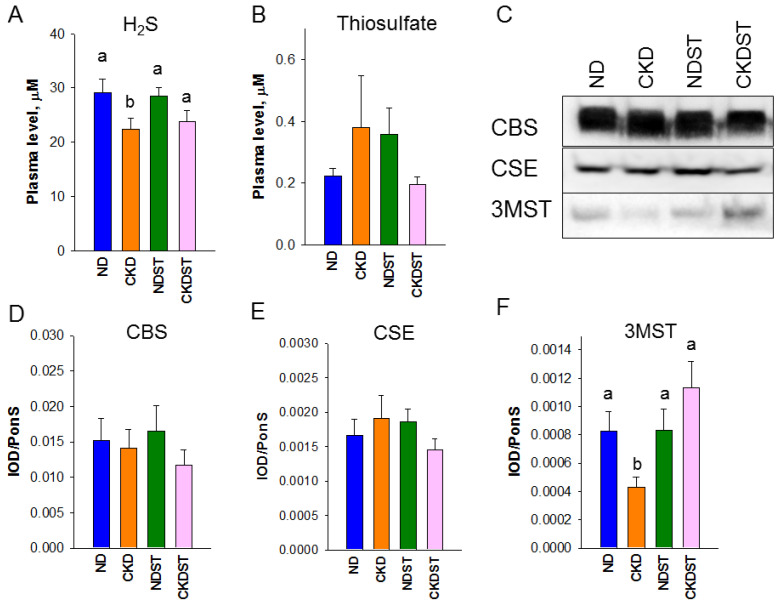
Plasma concentrations of (**A**) H_2_S and (**B**) thiosulfate, and renal protein abundance of H_2_S-producing enzymes. (**C**) Representative Western blot protein bands demonstrate immunoreactivity to CBS (61 kDa), CSE (45 kDa), and 3MST (52 kDa). Renal cortical protein abundance of (**D**) CBS, (**E**) CSE, and (**F**) 3MST was calculated. N = 8/group. Different letters above the column show significant differences between groups. CBS = cystathionine β-synthase; CSE = cystathionine γ-lyase; 3MST = 3-mercaptopyruvate sulfurtransferase.

To determine the influence of maternal CKD and STS administration on the H_2_S pathway, we determined plasma concentrations of H_2_S and thiosulfate and protein abundance of H_2_S-producing enzymes in the offspring’s kidneys (Figure 2). 

Male offspring in the CKD group exhibited plasma H_2_S concentration lower than controls at 12 weeks of age (Figure 2A), while plasma thiosulfate concentration was comparable among the four groups (Figure 2B). No differences were observed for renal protein levels of H_2_S-producing enzymes CBS and CSE among the four groups (Figure 2C). Nevertheless, exposure to maternal CKD diminished renal 3MST protein abundance, which was averted via maternal STS treatment (Figure 2F). Altogether, these observations reveal that the protective actions of STS treatment are relevant to increases in plasma H_2_S concentrations and the 3MST protein amount in the kidneys.

### 3.3. NO Pathway

**Figure 3:**
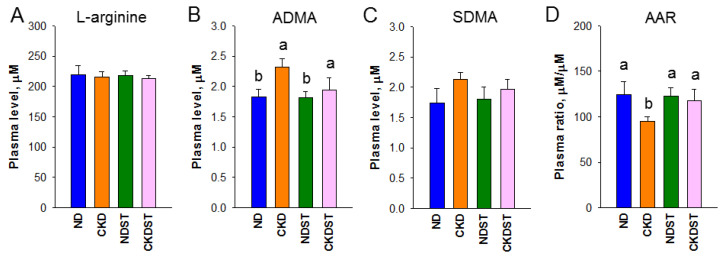
Plasma concentrations of nitric oxide (NO) parameters include (**A**) L-arginine, (**B**) asymmetric dimethylarginine (ADMA), (**C**) symmetric dimethylarginine (SDMA), and (**D**) L-arginine-to-ADMA ratio (AAR). N = 8/group. Different letters above the column show significant differences between groups.

As summarized in Figure 3, no differences in NO-related parameters were observed in terms of L-arginine and SDMA. Maternal CKD programming substantially increased plasma ADMA concentrations in adult offspring (Figure 3B). Additionally, maternal CKD reduced the L-arginine-to-ADMA ratio (AAR) in the CKD group (Figure 3D), which was prevented by means of maternal STS treatment. This observation, together with the fact that AAR represents NO bioavailability [17], suggests that STS protects adult offspring from hypertension and is possibly related to the restoration of NO.

### 3.4. Gut Microbiota Composition

**Figure 4:**
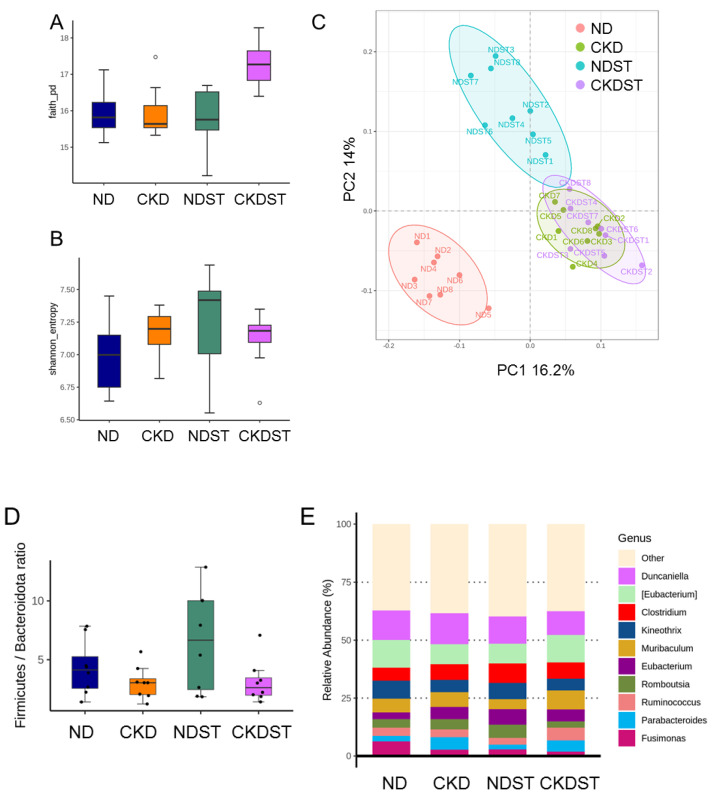
Box plots of (**A**) Faith’s phylogenetic diversity (PD) index and (**B**) Shannon index show alpha diversity in the gut microbiota of the four groups. (**C**) Principal coordinate analysis (PCoA) plots of beta diversity. Each data point represents one sample, and each color represents each group. (**D**) Variability in the *Firmicutes/Bacteroidetes* ratio in the gut microbiota. Each circle represents the data of a single sample. (**E**) 16s rRNA gene sequencing analysis of gut microbiota composition at the genus level.

Alpha diversity analysis was performed utilizing Faith’s PD index (Figure 4A) and the Shannon index (Figure 4B) to determine the species richness and evenness. Alpha diversity revealed that maternal CKD and STS have a negligible effect on each group. Beta diversity analysis (Figure 4C) was carried out utilizing PCoA plots to illustrate the phylogenetic distance of the bacterial communities of the fecal samples. The beta diversity analysis revealed that four groups had distinct clustering. However, the ND group samples were further apart. Additionally, ANOSIM revealed that the four groups differ greatly from each other (All *p* < 0.01). 

Consistent with prior animal studies [11,18], the major phyla are *Firmicutes* and *Bacteroidetes*, with subsequent *Deferribacteres* and *Actinobacteria*. The *Firmicutes*/*Bacteroidetes* (F/B) ratio was considered a microbial marker for hypertension [21]. Our data revealed the F/B ratio did not differ among the four groups (Figure 4D). At the genus level, the top ten dominant genera were comparable among the four groups (Figure 4E). 

**Figure 5:**
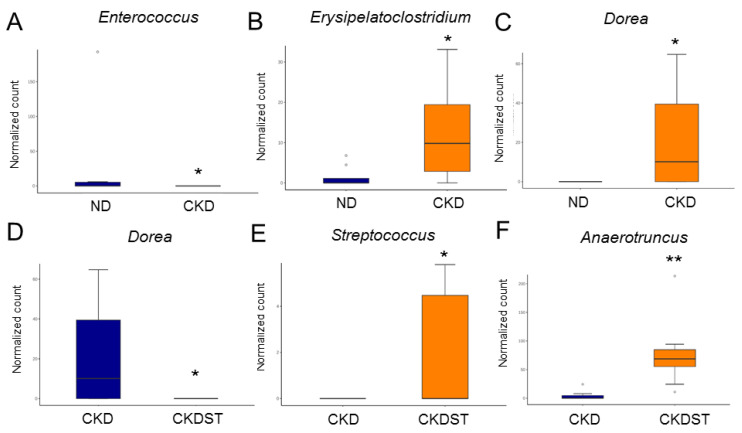
Composition of gut microbiota demonstrating different communities at the genus level. Relative abundance of (**A**) *Enterococcus*, (**B**) *Erysipelatoclostridium*, (**C**) *Dorea*, (**D**) *Dorea*, (**E**) *Streptococcus*, and (**F**) *Anaerotruncus*. * *p* < 0.05. ** *p* < 0.01.

Maternal CKD caused a decrease in genus *Enterococcus* and increases in genera of *Erysipelatoclostridium* and *Dorea* vs. the ND group (Figure 5A–C). Conversely, maternal CKD-induced reduction in genus *Dorea* was restored after STS treatment (Figure 5D). Compared with the CKD group, the abundance of genera *Streptococcus* and *Anaerotruncus* was higher in the CKDST group (Figure 5E,F). 

**Figure 6:**
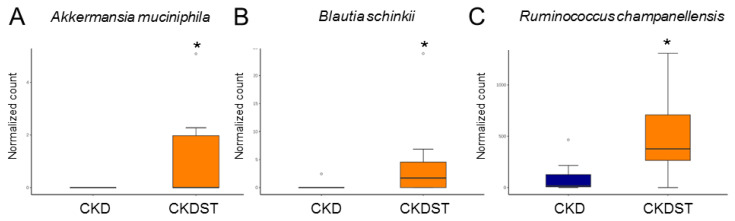
Composition of gut microbiota revealing different communities at the species level. Relative abundance of (**A**) *Akkermansia muciniphila*, (**B**) *Blautia schinkii*, and (**C**) *Ruminococcus champanellensis*. * *p* < 0.05.

To analyze the reasons for the protective effects of STS treatment and explore in more detail the gut microbiota component, we next illustrate the significant changes between the CKD and CKDST groups at the species level. We found that compared with the CKD group, *Akkermansia muciniphila* (Figure 6A)*, Blautia schinkii* (Figure 6B)*,* and *Ruminococcus champanellensis* (Figure 6C) were significantly increased in the CKDST group.

**Figure 7:**
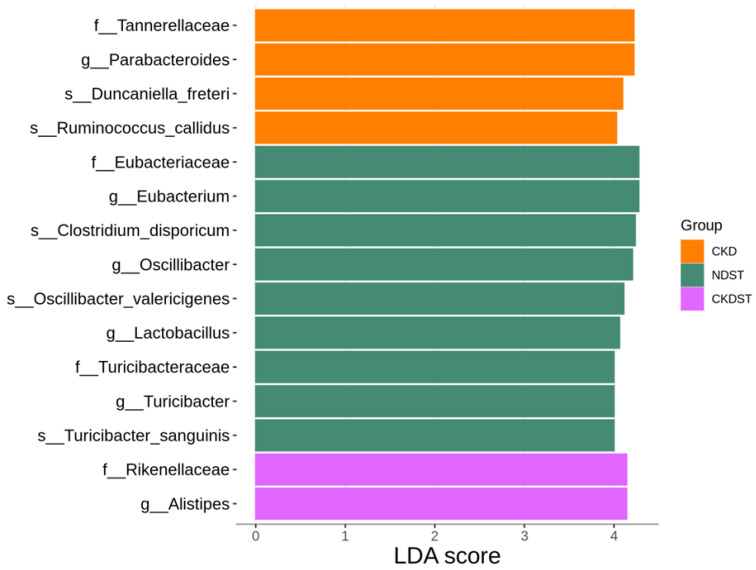
Linear discriminant analysis effect size (LEfSe) to identify the differentially abundant taxa between groups. It mainly shows the significantly different taxa with the linear discriminant analysis (LDA) score > 4. The color of the horizontal bar denotes the respective group.

LEfSe analysis was undertaken to further discover the differentially abundant taxa between groups (Figure 7). The CKD group exhibited a significant rise in the proportion of the genus *Parabacteroides*. STS treatment caused an increase in the genera *Eubacterium*, *Oscillibacter*, *Lactobacillus*, and *Turicibacter*. Additionally, LEfSe analysis identified the proportion of the genus *Alistipes* was augmented in the CKDST group. 

## 4. Discussion

**Figure 8:**
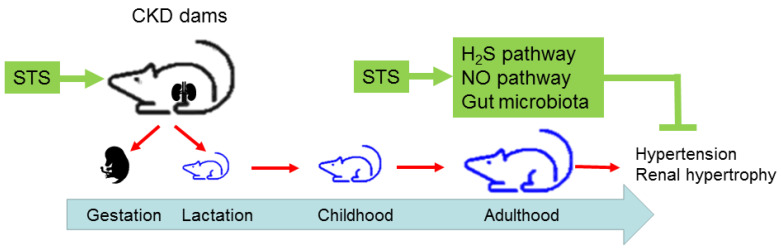
Schematic illustration of protective effects of sodium thiosulfate (STS) treatment and putative mechanisms underlying maternal chronic kidney disease (CKD)-induced offspring hypertension.

Our findings demonstrate that (i) maternal STS treatment prevented adult offspring from exhibiting hypertension induced by maternal CKD; (ii) treatment with STS during pregnancy and lactation restores maternal CKD-induced reduction of renal 3MST protein levels and plasma H_2_S concentration; (iii) the benefits of STS for offspring hypertension are connected to increased NO bioavailability; (iv) maternal treatment with STS alters microbiota beta diversity and composition in adult progeny; (v) maternal CKD reduced genus *Enterococcus* and increased genera of *Erysipelatoclostridium* and *Dorea*, while maternal STS treatment increased genus *Dorea*, *Streptococcus*, and *Anaerotruncus*; and (vi) the beneficial effect of STS against offspring hypertension coincided with increases of beneficial microbes such as *Akkermansia muciniphila*, *Blautia schinkii*, and *Ruminococcus champanellensis*. The protective effects and putative mechanisms are presented in Figure 8.

In support of prior research indicating that maternal illness results in long-term adverse offspring outcomes [8,9,10], we found that adult progeny born from CKD mothers developed hypertension, renal hypertrophy, and low body weight. That treatment with STS throughout gestation and lactation was able to improve offspring hypertension in a maternal CKD model is a novel finding. There was, however, no differential impact on body weight and the kidney-weight-to-body-weight ratio between the CKD and CKDST groups.

Though the anti-hypertensive effect of STS has been reported in CKD [13], our report goes beyond prior research and reveals maternal treatment with STS enables the prevention of offspring hypertension induced by maternal CKD. In most former studies, STS has been delivered via i.p. or i.v. administration. The novel observation that oral administration of STS exerts anti-hypertensive actions in the maternal CKD model offers opportunities for translation into clinical practice. 

Considering STS is a precursor of H_2_S [6], our observations are in line with prior work that supports the role played by H_2_S in the development of hypertension [22]. Importantly, H_2_S-related interventions, such as H_2_S donors and precursors of H_2_S, have shown preventive and therapeutic potential for adult diseases of developmental origins [23]. In the present study, the use of STS was ceased after weaning. Therefore, its actions are only due to reprogramming instead of direct effects. 

The beneficial actions of STS against maternal CKD-primed offspring hypertension might be associated with plasma H_2_S concentrations and increased renal 3MST protein abundance. Although oral administration of STS can directly increase urinary excretion of thiosulfate and sulfate [13], our results go beyond prior research showing that the use of STS in early life can have long-term effects on offspring’s H_2_S-generating system to increase H_2_S bioavailability later in life.

The BP-lowering effect of maternal STS treatment on adult offspring was achieved in the face of an increase in NO bioavailability. H_2_S is a physiological vasorelaxant through an enhancement of NO signaling [22]. This notion is supported by our data presenting that the beneficial action of STS was accompanied by decreased ADMA levels and increased AAR, a NO bioavailability index. Given that H_2_S has been proposed to exert an anti-oxidative effect against oxidative stress [1], and that impaired ADMA/NO pathway mediates oxidative stress implicating in hypertension [24], how the crosstalk between H_2_S and NO in the control of offspring’s BP is reprogrammed through STS treatment deserves further clarification. 

Another advantageous action of STS could be changes in gut microbiota composition. According to the available human and animal studies [25,26,27,28], genera *Streptococcus*, *Enterococcus*, *Anaerotruncus*, *Alistipes*, and *Eubacterium* were depleted, while genera *Parabacteroides*, *Dorea*, and *Erysipelatoclostridium* were enriched in hypertension. 

Consistent with previous reports, maternal CKD-induced offspring hypertension coincides with a high abundance of the genera *Parabacteroides*, *Dorea*, and *Erysipelatoclostridium*, and a low abundance of *Enterococcus*. Conversely, maternal STS treatment enriched several genera that are reported as negatively associated with BP, including *Streptococcus*, *Anaerotruncus*, and *Alistipes*. Importantly, maternal STS treatment increased the abundance of several beneficial microbes with potential probiotic properties, including *Akkermansia muciniphia* [29], *Blautia schinkii* [30], and *Ruminococcus champanellensis* [31]. Of note is that several studies have highlighted the positive role of *Akkermansia muciniphila* in improving hypertension [29]. To further understand the impact of STS on programmed hypertension, further research should be investigated to truly explore its actions on beneficial microbes and their interactions with BP regulation.

Moreover, we determined microbial taxa involved in sulfur metabolism. Our data indicated that all SRBs (e.g., *Desulfovibrio* or *Desulfobacter*) were not noticeable in both STS-treated groups. In the gut, several species with sulfite reductase can also participate in H_2_S production, including *E coli*, *Klebsiella*, *Bacillus*, *Corynebacterium*, *Salmonella*, *Rhodococcus*, etc. [32]. We observed that STS has a neglectable effect on the abundance of sulfite-reducing microbes. Therefore, it is not known whether the protective role of STS is connected to intestinal microbe-derived H_2_S and alterations of sulfite- or sulfate-reducing microorganisms.

In addition to gut microbiota dysbiosis, oxidative stress and inflammation also contribute to the pathogenesis of CKD and have been identified as molecular mechanisms for H_2_S effects [4,33]. Using the maternal CKD model, our previous study showed perinatal resveratrol therapy prevented offspring hypertension and is connected to the reduction of oxidative stress and an altered gut microbiome and microbe-derived metabolites [34]. Resveratrol, a natural polyphenol, exhibits antioxidant and anti-inflammatory properties. Considering the beneficial effect of STS in the present study, whether the application of nutraceuticals with anti-inflammatory or antioxidant properties could provide renoprotection and thereby avert maternal CKD-induced hypertension deserves to be investigated further.

Short-chain fatty acids (SCFAs) are the main microbiota-derived metabolites [35]. As we mentioned earlier, the beneficial effects of perinatal resveratrol therapy also contributed to the mediation of SCFA and their receptors [34]. Another study indicated that maternal CKD-induced offspring hypertension can be averted through the perinatal use of propionate, one of the predominant SCFAs [36]. Accordingly, targeting microbial metabolite SCFAs might also be an interesting mechanism to explore. Future studies should assess microbial metabolites and evaluate their connections with the protective actions of STS against maternal CKD-induced hypertension. 

Our study has a few limitations. Firstly, we did not assess the impact of STS treatment on sex differences, since only male progeny was used in the present study. Another limitation is that the microbiome data do not provide information on whether or not oral administration of STS during pregnancy and lactation could alter gut microbiota in mothers or neonate offspring. Whether STS treatment could regulate gut microbiota-derived fecal H_2_S connected to offspring hypertension awaits further clarification. Thirdly, we analyzed renal outcomes and gut microbiota in adult offspring at the time hypertension appeared, but not in dams. Our previous research indicated that adenine-fed mother rats displayed renal dysfunction, glomerular and tubulointerstitial damage, hypertension, and placental abnormalities [15]. Considering STS treatment has shown benefits against kidney disease and hypertension [12,13,14], additional research is needed to clarify whether STS treatment could also improve renal outcomes for mother rats. Whether STS treatment during pregnancy and lactation might alter the gut microbiota in both dams and offspring, and whether maternal alterations in renal outcomes and gut microbiota are connected with offspring outcomes, both require further evaluation. As several inflammatory mediators, such as NF-κB, NLRP3, and mitogen-activated protein kinase (MAPK) signaling pathways, were all activated in CKD and could represent a potential target for STS [37,38], studying these mechanisms might also be an interesting alternative target to explore. Lastly, the findings presented in our study are valuable for revealing that STS has beneficial effects on offspring programmed by maternal CKD but are limited to testing in this model. Further studies are needed in other animal models of CKD and humans before STS can be translated into clinical practice. Considering the significant progress that has been made over the last decade in RSS-related drugs [39,40,41], the reprogramming effects of other RSS-based interventions on maternal CKD-primed hypertension also deserve further attention.

## 5. Conclusions

To conclude, our results suggest that oral administration of STS during gestation and lactation improved offspring hypertension induced by maternal CKD via augmentation of both the H_2_S and NO pathways and changes in gut microbiota composition. As such, early-life intervention strategies specifically targeting the H_2_S signaling pathway could be considered for preventing hypertension in progeny born from mothers with CKD.

## Figures and Tables

**Table 1:** List of antibodies used for Western blot.

Antigen	Clonality	Source	Dilution
CSE	Polyclonal rabbit	Proteintech Group	1:1000
CBS	Monoclonal mouse	Abnova Corporation	1:1000
3MST	Monoclonal rabbit	Novus Biologicals	1:500

CSE = cystathionine γ-lyase; CBS = cystathionine β-synthase; 3MST = 3-mercaptopyruvate sulfurtransferase.

## Data Availability

The data that support the findings of this study are contained within the article.
